# Student Version of the Teacher–Student Relationship Inventory (S-TSRI): Development, Validation and Invariance

**DOI:** 10.3389/fpsyg.2020.01724

**Published:** 2020-07-28

**Authors:** Rebecca P. Ang, Soo Lin Ong, Xiang Li

**Affiliations:** ^1^National Institute of Education, Nanyang Technological University, Singapore, Singapore; ^2^Ministry of Education, Singapore, Singapore; ^3^Department of Applied Social Sciences, The Hong Kong Polytechnic University, Kowloon, Hong Kong

**Keywords:** teacher-student relationship, validation, academic achievement, school belonging, aggression

## Abstract

There is limited knowledge concerning children’s relationships with their teachers, and specifically, we lack a suitable, culturally appropriate measurement instrument for assessing the teacher-student relationship from the student’s perspective in Asia. This study used attachment theory as a theoretical framework to understand teacher-student relationships. Using a dataset from the Ministry of Education (MOE) of Singapore, the authors developed and validated a student version of the Teacher-Student Relationship Inventory (S-TSRI), with good psychometric properties for Singaporean children. The three-factor S-TSRI model comprising the factors satisfaction, instrumental help, and conflict was first established by exploratory factor analysis (EFA) and confirmed by confirmatory factor analysis (CFA). Through subsequent multigroup CFAs, we found that the factorial invariance was supported across gender, grade levels, and students of different academic levels, represented by the pass and fail groups. The structural model was tested in the total, pass, and fail groups. For the total and pass groups, the factors satisfaction and instrumental help showed significant positive relationships with a sense of school belonging, and negative or non-significant relationships with aggression. The conflict factor showed a weaker negative or non-significant relationship with a sense of school belonging, and a positive relationship with aggression. For the fail group, identical results were obtained with one exception; this was discussed in light of the fail group having a different needs profile. Findings from this study show that the 14-item S-TSRI measure has robust psychometric properties and yields scores that are reliable and valid in this large sample of primary school students from Singapore.

## Introduction

Teachers play a crucial role in the developmental trajectory of students, and a supportive teacher-student relationship is a very important determinant of students’ psychosocial and behavioral adjustment (Hughes et al., 1999; Ang, 2005; Mason et al., 2017). A meta-analysis based on 99 studies indicates that there are small to medium associations between the teacher-student relationship and academic achievement, and medium to large associations between the teacher-student relationship and students’ school engagement (Roorda et al., 2011). A supportive teacher-student relationship benefits children by fostering a positive sense of school belonging and promoting positive academic and behavioral outcomes (Roeser et al., 1996; Birch and Ladd, 1997; Mason et al., 2017). Compared with students who have a good relationship with teachers, students who have a poor teacher-student relationship have reported more difficulties in emotional and behavioral adjustment and higher levels of aggressive behavior (Milatz et al., 2014). In an academically at-risk sample of 706 primary school students, a positive teacher-student relationship was found to be related to students’ higher academic achievement, a greater sense of school belonging, and a lower level of externalizing behaviors (Wu et al., 2010).

### Attachment Theory and Teacher-Student Relationships

A core tenet of attachment theory (Bowlby, 1969) is that a strong and trusting bond between an infant and the caregiver is the basis for a child’s positive socioemotional development and emotional regulation. If the caregiver is responsive and sensitive, the infant will use the caregiver as a “safe base” from which to explore. According to Bowlby (1969), a child with secure attachment will develop an “internal working model” of how a mutually satisfying social relationship ought to be. These internal working models are mental representations that children use to guide behavior in developing successful social relationships with others in the future. The attachment perspective has been extended to understand teacher-student relationships as well.

In the extant literature, teachers have been regarded as “*ad hoc* attachment figures” (Zajac and Kobak, 2006; Verschueren and Koomen, 2012) from preschool to adolescence. Even though the role of the teacher as an attachment figure is expected to be of greater importance for younger children compared to older children, teachers remain as key figures in the lives of older children and adolescents. Students who have positive relationships with their teachers use teachers as a secure base to explore their classroom and school environment because they feel safe to do so. Additionally, they have an internal working model of a supportive and responsive relationship they experience. Consequently, these students are more willing to take on challenges, learn about socially appropriate behaviors, and develop their socioemotional skills (Hamre and Pianta, 2001). Teachers who allow the students enough independence for exploration, yet monitor and provide developmentally appropriate scaffolds, provide strong support for the growth of the child’s cognitive, social, and emotional competencies (Verschueren and Koomen, 2012). A close and supportive relationship with teachers, and one that is conflict-free, will serve as a “safe haven” and buffer from stress, permitting students to focus their energies on task engagement and interacting with significant others within the classroom and school context.

### Dimensions of Teacher-Student Relationships and Associations With Other Variables

In the literature, closeness and conflict are considered the two most common dimensions of the teacher-student relationship (Mason et al., 2017). The dimension of closeness is broadly defined as the degree to which a teacher-student relationship is a satisfactory and positive one, characterized by warmth, support, and affection. Some researchers have labeled this dimension as closeness (e.g., Pianta, 2001), whereas others have labeled it as satisfaction (e.g., Ang, 2005), support (Hughes et al., 2008), or warmth (Wu and Hughes, 2015). The dimension of conflict is defined as the degree to which a teacher-student relationship is a negative, unpleasant, and conflictual one. Across different researchers, this dimension appears to be consistently and universally labeled as conflict (Pianta, 2001; Ang, 2005; Hughes et al., 2008; Wu and Hughes, 2015).

Pianta (2001) found that in addition to closeness and conflict, dependency was a relevant dimension in the teacher-student relationship, especially for samples comprising younger children – either preschool children or those in the lower elementary school. Dependency is defined as the degree to which the student is clingy, overly dependent, and overly reliant on the teacher. For samples of older children in upper elementary school or in middle school, instrumental help would be a dimension of relevance in the teacher-student relationship. For students in elementary schools, teachers are resources who provide most instrumental help in addition to parents (Furman and Buhrmester, 1985). In their central role of transmitting knowledge and training to students, teachers provide information, advice, and instruction. Additionally, they model behavior and interact in ways to promote students’ social and academic development (Wentzel, 2009). Instrumental help is therefore defined as the degree to which teachers provide advice, encouragement, and have a caring attitude and a genuine interest in their students.

Effective teachers typically have close and satisfactory relationships with students, provide instrumental help, and care about students (Wentzel, 2009). The positive dimensions of the teacher-student relationship such as satisfaction, closeness, and instrumental help have consistently been found to be positively correlated with each other, and these have been found to be negatively correlated with negative dimensions of the teacher-student relationship such as conflict. For example, students’ satisfaction with teachers was positively related to teachers’ helping behavior and negatively related to teachers’ admonishing behavior (Kokkinos et al., 2009). In other studies, a close and satisfactory teacher-student relationship makes students pleased to see their teachers and less likely to have conflicts with teachers (Milatz et al., 2014; Cadima et al., 2015; Vervoort et al., 2015).

Different dimensions of the teacher-student relationship have also been found to be differentially associated with academic and behavioral outcomes. Early studies have found a significant association between teachers’ instrumental help and students’ engagement in the classroom (Skinner and Belmont, 1993). Subsequent studies continued to find that instrumental help increased students’ general interest in classroom activities and promoted their prosocial and compliant behaviors (Wentzel et al., 2010). Suldo et al. (2014) found that students who were satisfied with their teacher-student relationships performed better academically and held more positive attitudes toward teachers. Such students also had lower absenteeism and fewer internal and external problems. Moreover, a close and satisfactory teacher-student relationship was related to positive peer relationships, a stronger sense of school belonging and engagement, and reduced undisciplined behaviors (Cemalcilar, 2010; Hagenauer et al., 2015). However, in some studies, students’ aggression and conduct problems were unrelated to closeness with teachers (Glüer and Gregoriadis, 2016; Sette et al., 2016) or teachers’ warmth (Longobardi et al., 2016). Similarly, some studies did not find an association between teacher’s instrumental help and student’s aggressive behavior (Ang and Raine, 2009). Teacher-student conflicts have been documented to be related to significantly more problems such as aggressive and rule-breaking behavior (Ang and Raine, 2009; Milatz et al., 2014; Glüer and Gregoriadis, 2016; Sette et al., 2016). Conflict with teachers have been shown to be related to students’ lowered liking for school, participation in the classroom, academic commitment, and achievement (Birch and Ladd, 1997; Settanni et al., 2015). With respect to outcome variables, generally, having a stronger sense of school belonging is inversely related to violence or aggression (Resnick et al., 1997; Cemalcilar, 2010; Duggins et al., 2016).

In sum, the body of research evidence suggests that positive dimensions of teacher-student relationships (e.g., satisfaction, closeness, instrumental help) have consistently been related to positive outcomes such as school connectedness and a sense of school belonging while negative dimensions (e.g., conflict) of the teacher-student relationship have consistently been related to negative outcomes such as aggression or other behavioral problems. As reviewed, research findings concerning positive teacher-student relationship dimensions’ associations with negative outcomes and negative teacher-student relationship dimensions’ associations with positive outcomes have been more mixed and variable. In some studies, these variables have been reported to be unrelated to each other while in other studies, inverse relationships have been found. Finally, in the literature, results across studies show that school connectedness and belonging is negatively correlated with student aggression and violence.

Most studies focus on the teacher-student relationship in the general population, whereas few studies have examined these relationships specifically in students with low academic achievement. In general, students with higher academic achievement were more satisfied with the teacher-student relationship and had less conflict compared with those with lower academic achievement (Kokkinos et al., 2009; Sivan and Chan, 2013; Suldo et al., 2014). However, a positive teacher-student relationship is arguably even more crucial to students with academic problems or failure than it is to other students (Wu et al., 2010). Hughes and Kwok (2007) examined 443 low-achieving primary school students and found that a positive teacher-student relationship positively influenced students’ engagement at school. In a different study, Chong et al. (2010) investigated 523 low-achieving students and found that those who were satisfied with the relationships with their teachers had more positive attitudes toward teachers and school. Conflict with teachers negatively influenced the students’ attitudes toward both teachers and school.

### Measurement of Teacher-Student Relationships

The quality of the teacher-student relationship has typically been measured as a dyadic relationship, from the perspective of teachers. The Student-Teacher Relationship Scale (STRS) developed by Pianta (2001) includes dimensions such as closeness, dependency, and conflict, and is the most commonly used instrument measuring the quality of the teacher-student relationship in the existing literature. This scale has been validated in the United States (Pianta, 2001), Greece (Gregoriadis and Tsigilis, 2008), Netherlands (Koomen et al., 2012), Norway (Solheim et al., 2012), Turkey (Ogelmana and Seven, 2014), Germany and Austria (Milatz et al., 2014), Portugal and Belgium (Cadima et al., 2015), and Italy (Sette et al., 2016). Besides the STRS (Pianta, 2001), the other scale measuring dyadic teacher-student relationships from the teacher’s perspective that has received some empirical support internationally is the Teacher Student Relationship Inventory (T-TSRI; Ang, 2005). This is the only scale that was originally developed in an Asian context to measure the teacher-student relationship. The TSRI has dimensions such as satisfaction, instrumental help, and conflict, and has been used in Singapore (Ang and Raine, 2009; Chong et al., 2010; Huan et al., 2012), Australia (Kavenagh et al., 2012), and the United States (Suldo et al., 2014).

In the literature, there are far fewer scales to measure dyadic teacher-student relationships from the student’s perspective. One such measure is the Young Children’s Appraisals of Teacher Support (Y-CATS; Mantzicopoulos and Neuharth-Pritchett, 2003) which includes warmth/support, autonomy, and conflict, and is a measure to understand the student’s perception of their relations with teachers. This scale has been validated in the United States (Mantzicopoulos and Neuharth-Pritchett, 2003), Netherlands (Spilt et al., 2010), and Italy (Longobardi et al., 2016). Koomen and Jellesma (2015) developed the Student Perception of Affective Relationship with Teacher Scale (SPARTS) using a sample of upper elementary Dutch students, and the SPARTS has three dimensions, namely, closeness, conflict, and negative expectations. Another scale measuring the teacher-student relationship from the student’s perspective is the Questionnaire on Teacher Interaction (QTI; Wubbels et al., 1985). This scale measures the student’s views of the teacher’s behavior from two dimensions including influence (dominance-submission) and proximity (cooperation-opposition) across eight domains: leadership, understanding, helpful/friendly, uncertain, dissatisfied, student freedom, admonishing behavior, and strict. It has been validated in Turkey (Telli et al., 2007) and Indonesia (Maulana et al., 2012). Although there are a couple of scales measuring dyadic teacher-student relationships from the students’ perspective, these tend to be fairly lengthy. Many times, especially in educational settings, researchers need to administer a battery of measures within a relatively short available window, accommodating and adhering to school scheduling and constraints. Relatively brief and psychometrically robust measures are needed. Additionally, overall, most studies investigated the teacher-student relationships from teachers’ rather than students’ perspectives, and there are a very limited number of measures developed and validated for use in an Asian context.

### Current Study

Ang (2005) developed and validated a teacher version of the T-TSRI in Singapore. The T-TSRI is a self-report measure that assesses teachers’ perceptions of the quality of their relationships with students. Because the measurement of the teacher-student relationship in the field has generally used teachers’ perceptions (Birch and Ladd, 1997; Pianta, 2001), the aim of the current study was to develop a student version of the TSRI (S-TSRI), adapted from the T-TSRI, in order to examine teacher-student relationships from the perspective of students. There is a need to develop and validate a brief yet psychometrically robust measure for appropriate use in an Asian context. In educational settings, very often, we need different scales in a battery of instruments to measure a range of attitudes, emotions, behaviors, or experiences. This is time consuming and can easily result in respondent fatigue which is a well-documented phenomenon. Hence, brief yet psychometrically robust scales are particularly valuable.

We hypothesized that the three factors, satisfaction, instrumental help, and conflict, can be extracted from the S-TSRI (Hypothesis 1). Research has shown that academically successful adolescents value instrumental help from teachers, as well as teachers’ warmth and acceptance (Wentzel, 2009). However, few studies have examined the perceptions that students with low academic achievement have of the teacher-student relationship, which was examined in the present study. We examined students of differing academic achievements, operationalized in this study as students who passed the English language examination versus those who failed. We tested the invariance of the 3-factor model of the teacher-student relationship, hypothesizing that the factor structure will be invariant across the pass and fail groups (Hypothesis 2). After validating the factor structure of the teacher-student relationship, we tested four hypotheses (Hypotheses 3–6) across all three samples: the total sample, the sample comprising students who passed the English language examination, and the sample comprising students who failed the English language examination. Specifically, we investigated the relationships among the positive (satisfaction, instrumental help) and negative (conflict) teacher-student relationship dimensions, and various outcomes, such as the students’ sense of school belonging and also their aggressive behavior. We expected satisfaction and instrumental help to correlate positively with each other and negatively with conflict (Hypothesis 3). We expected satisfaction and instrumental help to have significant positive associations with a sense of school belonging, and weaker negative or non-significant associations with aggression (Hypothesis 4). We expected conflict to have a significant positive association with aggression and a weaker negative or non-significant association with a sense of school belonging (Hypothesis 5). We expected a sense of school belonging to be negatively correlated with aggression (Hypothesis 6).

## Materials and Methods

### Scale Development

A total of 47 items about various aspects of the quality of teacher-student relationships were generated based on a literature review in this domain, adapted items from the T-TSRI, and focus group interviews with teachers and primary school students. The development of this scale was anchored on attachment theory. Just as a child with secure attachment will likely develop an “internal working model” of how mutually satisfying social relationships will be, teachers often serve as compensatory resources for students, especially students who are demographically and behaviorally at risk. Having a satisfactory, supportive, and conflict-free teacher-student relationship, and one in which teachers provide sufficient instrumental help to students, will permit students to view this relationship as a secure base from which they can safely explore their environment academically and socioemotionally. Therefore, the items generated and refined for this scale were built upon the tenets of attachment theory. Relevant items from the T-TSRI were adapted to reflect the perspective of students instead of teachers, and new items reflecting key considerations in the quality of the teacher-student relationship from the students’ perspective were also included. We conducted 12 focus group interviews with Primary 4 students from six primary schools with an average of eight students in each focus group. The students provided feedback on the item wording, language used, and comprehensibility of these items. Likewise, focus group interviews with the teachers also provided an opportunity to receive feedback from the teachers so as to ensure that the items tapped into relevant content and domain areas. Additionally, the focus group interviews conducted with teachers and primary school students provided helpful feedback to further refine the language and phrasing of these items so that they were developmentally and culturally appropriate. Duplicate and problematic items were removed resulting in a pool of 40 items. Students were asked to think about their form teacher when they responded to the 40 items. The form teacher whom the students are providing ratings on with respect to the teacher-student relationship was not present in the classroom when the survey was administered. Students rated the 40 items on the extent to which they agreed with each statement with respect to their form teacher at the end of the school year, using a 5-point Likert scale (1 = *almost never true*, 2 = *seldom true*, 3 = *sometimes true*, 4 = *often true*, and 5 = *almost always true*). A form teacher in the Singapore school system is a teacher who has the responsibility to take care of a particular class in a school. In primary schools in Singapore, there is dedicated time within the curriculum for form teachers to engage in quality interactions with their students and for them to help the students strengthen their social and emotional competencies. Hence, students generally know their form teachers well.

### Participants

The data were from a total of 6,578 students (48.5% males, *n* = 3,190; 51.5% females, *n* = 3,388) from 18 randomly selected government primary schools, across three grade levels in Singapore: Primary 4 (34.2%; *n* = 2,247), Primary 5 (33.2%; *n* = 2,186), and Primary 6 (32.6%; *n* = 2,145). Students’ self-reported ethnicities were as follows: Chinese (69.0%; *n* = 4,538), Malay (17.1%; *n* = 1,126), Indian (9.5%; *n* = 622), and other ethnic groups (4.4%; *n* = 290), and this distribution approximately mirrors the larger Singapore census data.

### Measures

#### Student Version of the Teacher Student Relationship Inventory (S-TSRI)

An initial 40 items tapping into different aspects of the quality of teacher-student relationships from the students’ perspective were included. To develop and validate this measure, we subsequently performed exploratory and confirmatory factor analyses. This resulted in a final 14-item S-TSRI measure consisting of three factors: Satisfaction (5 items; e.g., “I am happy with my relationship with this teacher”), Instrumental help (5 items; e.g., “If I need someone to listen to me, I will go to this teacher”), and Conflict (4 items; e.g., “If this teacher is absent, I feel relieved”), rated on a 5-point scale from 1 (*almost never true*) to 5 (*almost always true*). A higher score in these dimensions indicates a higher level of satisfaction, instrumental help, and conflict with teachers, respectively. The present sample’s Cronbach alpha for Satisfaction, Instrumental help, and Conflict were good at 0.90, 0.86, and 0.85, respectively.

#### Psychological Sense of School Membership Scale (PSSM)

The 18-item PSSM (Goodenow, 1993) provided a self-report of students’ sense of school belonging (e.g., “I am included in lots of activities at this school”). Five items (e.g., “Sometimes I feel as if I don’t belong in this school”) were reverse scored. All items were rated on a 5-point scale ranging from 1 (*strongly disagree*) to 5 (*strongly agree*), with higher scores indicating a stronger sense of belonging in school. The present sample’s Cronbach alpha for the PSSM was good at 0.87.

#### Aggression Questionnaire (AQ)

The 12-item AQ (Buss and Warren, 2000) provided a self-report of students’ aggressive behavior. Students rated the description of each item (e.g., “I have threatened people I know”) on a scale from 1 (*not at all like me*) to 5 (*completely like me*). A higher score suggested a higher level of aggression. The present sample’s Cronbach alpha for aggression was good at 0.85.

#### English Language Examination Score

Students’ end-of-the-year English language examination score was also obtained for this study. English language was selected because it is a subject that is common across different grade levels and schools in Singapore. English is used as the medium of instruction for all subjects (with the exception of Mother Tongue languages) in the Singapore education system. In this study, we tested for invariance across the pass/fail groups. A score of 50 out of 100 marks is considered a passing score; on this basis, students who scored higher than or equal to 50 and lower than 50 were classified into the pass and fail groups, respectively. Of the 6,466 students in the database with reported English scores, 5,851 students passed the year-end English language examination and 615 students failed.

### Data Analytic Plan

First, exploratory and confirmatory factor analyses (EFA and CFA) were performed to examine the factor structure of the student version of the Teacher-Student Relationship Inventory (S-TSRI). In preparation for the EFA and CFA, the full sample was randomly divided into two halves: Sample A (*n* = 3,289) and Sample B (*n* = 3,289). EFA was used to explore the factor structure of the S-TSRI on Sample A using SPSS 23.0, and CFA was used to confirm the factor structure obtained from EFA using LISREL 8.7 (Jöreskog and Sörbom, 2004) on Sample B. For EFA, principal component analysis (PCA) with varimax rotation was used. We based the decision about number of factors to retain on a combination of methods including eigenvalue >1.0, scree plots, as well as conceptual clarity, theoretical salience of the factors, and simple structure. Our goal was to have the smallest number of possible factors and for each item to load on only one latent factor. Items should preferably load greater than 0.40 on the relevant factor and less than 0.40 on all other factors (Stevens, 1996). For CFA, we tested null, one-factor, two-factor, and three-factor models with an evaluation of a series of model fit indices including the comparative fit index (CFI), the incremental fit index (IFI), the non-normed fit index (NNFI), and the root mean square error of approximation (RMSEA). CFI, IFI, and NNFI ≥ 0.95 are considered to be a superior fit; and RMSEA ≤ 0.06 is indicative of a good fit (Hu and Bentler, 1999).

Second, students were separated into the pass or fail group based on the passing score of 50 marks out of 100. The S-TSRI model that was established by EFA and confirmed by CFA was first tested in the two groups separately, and then the measurement and structural invariance were tested across the two groups by conducting a series of progressively restrictive invariance models. A model with no equality constraints was first established, followed by six models constraining the factor loadings, item intercepts, error variances, factor variances, factor covariances, and factor means equally (Vandenberg and Lance, 2000). Similarly, we performed measurement and structural invariance tests for gender and grade levels. Because of the over-sensitivity of Δχ^2^ to sample size, ΔCFI ≤ 0.01 (Cheung and Rensvold, 2002) and RMSEA ≤ 0.015 (Chen, 2007) were used to indicate invariance.

Third, the relationships between the dimensions of the S-TSRI, the sense of school belonging, and aggressive behavior were tested by structural equation modeling (SEM). Specifically, the two-step modeling approach (Anderson and Gerbing, 1988) was employed: the measurement model is essentially the CFA that examines the relationships between the latent constructs and their observed indicators, while the structural model examines the inter-correlations among the latent constructs (Schreiber et al., 2006). To evaluate the goodness of fit of the model, various indices (i.e., CFI, IFI, NNFI, and RMSEA) were used. Finally, the established structural model was examined separately in the pass and fail groups. Relations between S-TSRI and English language examination scores were also tested for the total sample, and pass and fail groups.

### Ethical Approval

Ethical approval for this study was granted by the Institutional Review Board of Nanyang Technological University, Singapore. There were no personal identifiers in this dataset and the study authors do not have access to any information that could lead to the data being linked to specific students.

### Procedure

The de-identified data for this study were obtained from an archival dataset from the Ministry of Education, Singapore. De-identified data comprised relevant information for the purposes of this study including demographics, students’ year-end English language examination score, and student ratings of the quality of teacher-student relationships, a sense of school belonging, and aggressive behavior. As English is the medium of instruction in the Singapore education system, all relevant information for the study was in English. Students had previously completed the survey in a classroom setting. The class form teacher was not present in the classroom when the survey was administered. Instead, an administrator was present to oversee the survey administration in the classrooms.

## Results

### Exploratory Factor Analysis (EFA) and Confirmatory Factor Analysis (CFA)

Prior to CFA, we first performed an EFA using Sample A. The value of the Kaiser-Meyer-Olkin (KMO) was 0.937 and Bartlett’s test of sphericity was significant: χ2 (91, *n* = 3,289) = 25,484, ρ < 0.001, suggesting that the data were appropriate to proceed with factor analysis. Of the 40 items, a total of 26 items were dropped from subsequent analyses because these items either had very low communalities, loaded greater than 0.40 on multiple factors, or did not have a factor loading of at least 0.40 on any factor. These procedures resulted in a 3-factor, 14-item measure accounting for a total of 68.32% of the variance in S-TSRI scores. The three factors were labeled Satisfaction, Instrumental help, and Conflict. Specifically, items 1–5 reflect Satisfaction, items 6–10 reflect Instrumental help, and items 11–14 reflect Conflict (see Table 1).

**TABLE 1:** Exploratory factor analysis (EFA) and confirmatory factor analysis (CFA) of the S-TSRI.

				3-factor Factor Loadings					
				
				EFA (*n* = 3,289)			CFA (*n* = 3,289)		
					
				Sat	Help	Con	Sat	Help	Con
1. I enjoy attending the class of this teacher.				0.71			0.78		
2. My relationship with this teacher is positive.				0.76			0.74		
3. If this teacher retires or leaves the school, I will miss him/her.				0.71			0.78		
4. I am happy with my relationship with this teacher.				0.81			0.82		
5. I like this teacher.				0.79			0.85		
6. If I have a problem at home, I will ask this teacher for help.					0.81			0.72	
7. I share about my personal life with this teacher.					0.78			0.72	
8. If I need help, I will go to this teacher.					0.68			0.77	
9. If I need someone to listen to me, I will go to this teacher.					0.81			0.82	
10. I depend on this teacher for advice.					0.65			0.71	
11. This teacher frustrates me more than other teachers who teach my class.						0.77			0.66
12. I cannot wait for this year to be over because I do not want to be taught by this teacher again.						0.77			0.83
13. If this teacher is absent, I feel relieved.						0.77			0.79
14. If I am not taught by this teacher, I will be able to enjoy my class more.						0.79			0.80

**Models**	**SB*χ^2^***	***df***	**CFI**	**IFI**	**NNFI**	**RMSEA (90% *CI*)**	**Compare**	**ΔSB*χ^2^***	**Δ*df***

Null	47424	91	0.252	0.252	0.252	0.398 (0.395–0.401)			
1-factor	6710	77	0.895	0.895	0.876	0.162 (0.159–0.165)	1 vs. 0	40714***	14
2-factor	4237	76	0.934	0.934	0.921	0.129 (0.126–0.132)	2 vs. 1	2473***	1
3-factor	796	74	0.989	0.989	0.986	0.054 (0.051–0.058)	3 vs. 2	3441***	2

SBχ*^2^*, Satorra-Bentler chi-square; *df*, degrees of freedom; CFI, comparative fit index; IFI, incremental fit index; NNFI, non-normed fit index; RMSEA, root mean square error of approximation; CI, confidence interval. ***p < 0.001.

Based on the EFA results obtained, we used Sample B to conduct a CFA to confirm the three-factor structure of the S-TSRI. Instead of merely confirming the three-factor structure in CFA, we tested null, one-factor, two-factor, and three-factor models to provide evidence that the three-factor structure would indeed have the best relative fit in comparison to the others. The one-factor model comprised all 14 items loading on a single factor. The two-factor model comprised one factor representing the positive dimension (Satisfaction/Instrumental help: 10 items) of the TSR, and the other factor representing the negative dimension (Conflict: 4 items) of the TSR. The three-factor model comprised 5 items loading on the Satisfaction factor, 5 items loading on the Instrumental help factor, and 4 items loading on the Conflict factor as derived from the EFA. All model fit indices confirmed the superiority of the three-factor model over the other models (see Table 2): SBχ^2^ (74, *n* = 3,289) = 796, *p* < 0.001, CFI = 0.989, IFI = 0.989, NNFI = 0.986, and RMSEA = 0.054 (90% *CI*:0.051–0.058). The latent factor correlations were as follows: Satisfaction and Instrumental help, *r* = 0.70 (*p* < 0.001); Satisfaction and Conflict, *r* = −0.74 (*p* < 0.001); and Instrumental help and Conflict, *r* = −0.46 (*p* < 0.001).

**TABLE 2:** Testing for pass/fail invariance: Results of multigroup CFA on S-TSRI.

	Overall fit indices							Comparative fit indices			
		
Model	SBχ^2^	*df*	CFI	IFI	NNFI	RMSEA (90% *CI*)	Compare	ΔSBχ*^2^*	Δ*df*	ΔCFI	ΔRMSEA
1	1441	148	0.990	0.990	0.987	0.052 (0.050–0.055)					
2	1501	159	0.989	0.989	0.988	0.051 (0.049–0.054)	2 vs. 1	60***	11	0.001	0.001
3	1637	170	0.988	0.988	0.988	0.052 (0.049–0.054)	3 vs. 2	136***	11	0.001	0.001
4	1765	184	0.988	0.988	0.988	0.052 (0.049–0.054)	4 vs. 3	128***	14	0.000	0.000
5	1775	187	0.987	0.987	0.988	0.051 (0.049–0.053)	5 vs. 4	10*	3	0.001	0.001
6	1820	190	0.987	0.987	0.988	0.052 (0.049–0.054)	6 vs. 5	45***	3	0.000	0.001
7	1909	193	0.986	0.986	0.987	0.052 (0.050–0.055)	7 vs. 6	89***	3	0.001	0.000

Model 1: configural invariance; Model 2: metric invariance; Model 3: scalar invariance; Model 4: error variance invariance; Model 5: factor variance invariance; Model 6: factor covariance invariance; Model 7: factor mean invariance. *p < 0.05, ***p < 0.001.

### Invariance Across the Pass Group and Fail Group

To test the invariance of the factor structure, the independent best-fit models for the pass and fail groups were established first (Byrne, 2006). We found that the three-factor model was good for the pass group: SBχ^2^(74, *n* = 5,851) = 1,260, *p* < 0.001, CFI = 0.990, IFI = 0.990, NNFI = 0.988, and RMSEA = 0.052 (90% *CI*:0.050–0.055); and the fail group: SBχ^2^(74, *n* = 615) = 164, *p* < 0.001, CFI = 0.988, IFI = 0.988, NNFI = 0.985, and RMSEA = 0.044 (90% *CI*:0.035–0.054). Subsequently, seven progressively restrictive invariance models were run to test the invariance of the three-factor structure of the S-TSRI across the pass group and fail group (see Table 2). ΔCFI ≤ 0.001 and ΔRMSEA ≤ 0.001 were indicative of the invariance of the two groups.

### Invariance Across Gender

To examine the invariance of the factor structure, the independent best-fit models for the male and female groups were established first (Byrne, 2006). We found that the three-factor model was good for the male group: SBχ^2^(74, *n* = 3,190) = 582, *p* < 0.001, CFI = 0.991, IFI = 0.991, NNFI = 0.989, and RMSEA = 0.046 (90% *CI*:0.043–0.050); and the female group: SBχ^2^(74, *n* = 3,388) = 851, *p* < 0.001, CFI = 0.988, IFI = 0.988, NNFI = 0.986, and RMSEA = 0.056 (90% *CI*:0.052–0.059). Likewise, we performed seven progressively restrictive invariance models to test the invariance of the three-factor structure of the S-TSRI across gender (see Table 3). ΔCFI ≤ 0.001 and ΔRMSEA ≤ 0.001 provided evidence of gender invariance.

**TABLE 3:** Testing for gender invariance: Results of multigroup CFA on S-TSRI.

	Overall fit indices							Comparative fit indices			
		
Model	SBχ^2^	*df*	CFI	IFI	NNFI	RMSEA (90% *CI*)	Compare	ΔSBχ*^2^*	Δ*df*	ΔCFI	ΔRMSEA
1	1434	148	0.990	0.990	0.987	0.051 (0.049–0.054)					
2	1486	159	0.989	0.989	0.988	0.050 (0.048–0.053)	2 vs. 1	52***	11	0.001	0.001
3	1606	170	0.989	0.989	0.988	0.051 (0.048–0.053)	3 vs. 2	120***	11	0.000	0.001
4	1810	184	0.987	0.987	0.987	0.052 (0.050–0.054)	4 vs. 3	204***	14	0.002	0.001
5	1869	187	0.987	0.987	0.987	0.052 (0.050–0.055)	5 vs. 4	59***	3	0.000	0.000
6	1931	190	0.986	0.986	0.987	0.053 (0.051–0.055)	6 vs. 5	62***	3	0.001	0.001
7	2050	193	0.985	0.985	0.986	0.054 (0.052–0.056)	7 vs. 6	119***	3	0.001	0.001

Model 1: configural invariance; Model 2: metric invariance; Model 3: scalar invariance; Model 4: error variance invariance; Model 5: factor variance invariance; Model 6: factor covariance invariance; Model 7: factor mean invariance. ***p < 0.001.

### Invariance Across Grade Level

To test the invariance of the factor structure, the independent best-fit models for different grade levels were established first (Byrne, 2006). We found that the three-factor model had a good fit for Primary 4: SBχ^2^(74, *n* = 2,247) = 369, *p* < 0.001, CFI = 0.991, IFI = 0.991, NNFI = 0.989, and RMSEA = 0.042 (90% *CI*:0.038–0.047); Primary 5: SBχ^2^(74, *n* = 2,186) = 538, *p* < 0.001, CFI = 0.989, IFI = 0.989, NNFI = 0.986, and RMSEA = 0.054 (90% *CI*:0.049–0.058); and Primary 6: SBχ^2^(74, *n* = 2,145) = 574, *p* < 0.001, CFI = 0.990, IFI = 0.990, NNFI = 0.987, and RMSEA = 0.056 (90% *CI*:0.052–0.061). Likewise, seven progressively restrictive invariance models were conducted to test the invariance of the three-factor structure of the S-TSRI across the grade levels (see Table 4). ΔCFI ≤ 0.001 and ΔRMSEA ≤ 0.001 provided evidence of invariance across the three grade levels.

**TABLE 4:** Testing for grade level invariance: Results of multigroup CFA on S-TSRI.

	Overall fit indices							Comparative fit indices			
		
Model	SBχ^2^	*df*	CFI	IFI	NNFI	RMSEA (90% *CI*)	Compare	ΔSBχ*^2^*	Δ*df*	ΔCFI	ΔRMSEA
1	1474	222	0.990	0.990	0.988	0.051 (0.048–0.053)					
2	1547	244	0.990	0.990	0.988	0.049 (0.047–0.052)	2 vs. 1	73***	22	0.000	0.002
3	1894	266	0.987	0.987	0.987	0.053 (0.051–0.055)	3 vs. 2	347***	22	0.003	0.004
4	2175	294	0.985	0.985	0.986	0.054 (0.052–0.056)	4 vs. 3	281***	28	0.002	0.001
5	2205	300	0.985	0.985	0.986	0.054 (0.052–0.056)	5 vs. 4	30***	6	0.000	0.000
6	2250	306	0.984	0.986	0.986	0.054 (0.052–0.056)	6 vs. 5	45***	6	0.001	0.000
7	2327	312	0.984	0.984	0.986	0.054 (0.052–0.056)	7 vs. 6	77***	6	0.000	0.000

Model 1: configural invariance; Model 2: metric invariance; Model 3: scalar invariance; Model 4: error variance invariance; Model 5: factor variance invariance; Model 6: factor covariance invariance; Model 7: factor mean invariance. ***p < 0.001.

### Measurement Model and Structural Model

The measurement model included five latent constructs: Satisfaction (5 observed indicators), Instrumental help (5 observed indicators), Conflict (4 observed indicators), PSSM (18 observed indicators), and aggressive behavior (12 observed indicators). The results of the measurement model show a good model fit: SBχ^2^(892, *n* = 6,578) = 14,025, *p* < 0.001, with CFI of 0.965, IFI of 0.965, NNFI of 0.963, and RMSEA of 0.047 (90% *CI*: 0.047–0.048). The factor loadings ranged from 0.76 to 0.86 for Satisfaction, 0.71 to 0.81 for Instrumental help, 0.67 to 0.82 for Conflict, 0.21 to 0.67 for PSSM, and 0.25 to 0.68 for aggressive behavior. The factor loadings of all observed indicators on the latent constructs were significant (*p* < 0.001), suggesting that all latent constructs were well represented by their observed indicators. The five latent constructs were significantly correlated with each other (see Table 5).

**TABLE 5:** Correlations among latent constructs of the S-TSRI, school belonging, and aggression.

	1	2	3	4	5
1. Satisfaction	–				
2. Help	0.69***	–			
3. Conflict	−0.73***	−0.47***	–		
4. School Belonging	0.51***	0.51***	−0.40***	–	
5. Aggression	−0.23***	−0.17***	0.32***	−0.40***	–

***p < 0.001.

Prior to investigating the paths in the structural model, we report the descriptive statistics for the study variables for the total sample as well as pass and fail groups. The means and standard deviations of the three dimensions of the S-TSRI, PSSM, and aggression are presented in Table 6. The independent *t*-tests suggest that students who passed the year-end English language examination report more satisfaction with their relationships with teachers (*t* = −4.74, *p* < 0.001), had a stronger sense of belonging in school (*t* = −9.31, *p* < 0.001), and had a much higher English language examination score (*t* = −94.94, *p* < 0.001) than students who failed the year-end examination. On the other hand, students who failed the year-end English language examination reported more conflicts with their teachers (*t* = 9.30, *p* < 0.001) and displayed more aggressive behavior (*t* = 8.00, *p* < 0.001) than students who passed the year-end examination. It was noted that both the pass and fail group students reported a similar level of instrumental help from their teachers (*t* = 1.36, *p* > 0.05).

**TABLE 6:** Descriptive analyses of the S-TSRI, school belonging, and aggression in all students, pass group, and fail group.

Variables	All		Pass		Fail		
			
	Mean	SD	Mean	SD	Mean	SD	*t*-test
Satisfaction	19.83	4.55	19.95	4.52	19.04	4.66	−4.74***
Help	14.23	5.32	14.19	5.34	14.50	5.16	1.36
Conflict	7.52	3.95	7.33	3.88	8.96	4.14	9.30***
School Belonging	64.40	11.92	64.90	11.98	60.67	10.58	−9.31***
Aggression	27.45	9.36	27.07	9.20	30.45	10.07	8.00***
English	69.45	14.07	72.39	11.06	41.46	7.24	−94.94***

Group 0 = fail; Group 1 = pass. ***p < 0.001.

**FIGURE 1:**
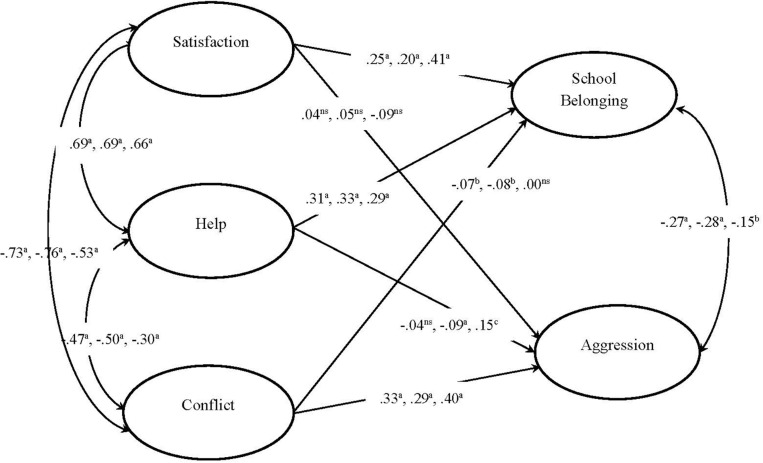
Structural equation model. Latent constructs are shown in ellipses. The standardized coefficients of all paths from left to right represent the entire, pass, and fail samples, respectively, a = *p* < 0.001; b = *p* < 0.01; c = *p* < 0.05; ns = non-significant.

To investigate our hypotheses, a structural model was examined. The structural model (see Figure 1) fit the data well: SBχ^2^(892, *n* = 6,578) = 14,025, *p* < 0.001, with CFI of 0.965, IFI of 0.965, NNFI of 0.963, and RMSEA of 0.047 (90% *CI*: 0.047–0.048). Results showed that the positive dimensions of TSR such as Satisfaction (β = 0.25, *p* < 0.001) and Instrumental help (β = 0.31, *p* < 0.001) strongly predicted school belonging, but Satisfaction (β = 0.04, *p* > 0.05) and Instrumental help (β = −0.04, *p* > 0.05) were unrelated to aggression. Conversely, the negative dimension of TSR, Conflict, was strongly related to aggression (β = 0.33, *p* < 0.001) but had a much weaker inverse relation to school belonging (β = −0.07, *p* < 0.01). Both Satisfaction and Instrumental help correlated positively with each other (β = 0.69, *p* < 0.001) but negatively with Conflict (Conflict and Satisfaction: β = −0.73, *p* < 0.001; Conflict and Instrumental help: β = −0.47, *p* < 0.001). School belonging and aggression were negatively correlated (β = −0.27, *p* < 0.001).

The structural model was then tested in the pass and fail groups separately (see Figure 1). Similarly, the model had a good fit for the pass group: SBχ^2^(892, *n* = 5,851) = 12,558, *p* < 0.001, with CFI of 0.967, IFI of 0.967, NNFI of 0.965, and RMSEA of 0.047 (90% *CI*: 0.047–0.048). The patterns of relationships for the pass group mirrored the results for the total sample. The structural model also had a good fit with the fail group: SBχ^2^(892, *n* = 615) = 2,125, *p* < 0.001, with CFI of 0.950, IFI of 0.950, NNFI of 0.947, and RMSEA of 0.047 (90% *CI*: 0.045–0.050). The patterns of relationships for the fail group were largely similar to those of the pass group and the total sample with a couple of exceptions. Conflict was not related to school belonging (β = 0.00, *p* > 0.05), and while this differed slightly from the pattern of findings for the total sample and pass group, this was in line with our hypothesis. The unexpected finding was that Instrumental help correlated positively with aggression (β = 0.15, *p* < 0.05) in the fail group.

Additionally, correlation analyses between TSRI dimensions (Satisfaction, Instrumental Help, Conflict) and the English language examination scores were performed. Students who reported more satisfaction with their relationships with teachers had higher English language examination scores (total sample *r* = 0.10, *p* < 0.01; pass group *r* = 0.09, *p* < 0.01; fail group *r* = 0.09, *p* < 0.05). Students who reported greater conflict with their teachers had lower English language examination scores (total sample *r* = −0.15, *p* < 0.01; pass group *r* = −0.09, *p* < 0.01; fail group *r* = −0.19, *p* < 0.01). Students’ perception of instrumental help from their teachers was found to be unrelated to students’ English language examination scores (total sample *r* = −0.02, *p* > 0.05; pass group *r* = −0.01, *p* > 0.05; fail group *r* = 0.02, *p* > 0.05).

## Discussion

This study aimed to develop a TSRI instrument examining the teacher-student relationship from the student’s perspective, appropriate for use in an Asian context. Findings from EFA suggested that the three factors of satisfaction, instrumental help, and conflict, can be extracted from the S-TSRI scores. This three-factor structure was confirmed via CFA in an independent sample, with the S-TSRI scales (Satisfaction, Instrumental Help, Conflict) showing evidence that the scores were internally consistent, with strong and satisfactory Cronbach alpha estimates, providing support for Hypothesis 1. Additionally, we tested competing models beyond the hypothesized three-factor model and evidence showed that the three-factor structure had the best fit with the data in comparison with the null, one-factor, and two-factor models. While the EFA approach plays a crucial role in the scale development and validation process, it cannot be used exclusively as a basis for a final determination regarding an underlying construct (Gorsuch, 1983; Thompson and Daniel, 1996). In the present study, both EFA and CFA procedures were used and rival models tested, thereby providing researchers with stronger evidence of the validity of the S-TSRI measure (Thompson, 2004).

As the S-TSRI is intended to be administered in a heterogeneous population of students with varying levels of academic achievement, it would be important to establish that the S-TSRI’s measurement properties are invariant across subgroups of the population. The present research investigated the measurement invariance of the three-factor structure of the S-TSRI across pass and fail groups of students using multigroup CFA. Results indicated configural, metric, scalar, error variance, factor variance, factor covariance, and factor mean invariance, providing support for Hypothesis 2. Put differently, this means that students from both the pass and fail groups in this Singapore sample interpreted the S-TSRI in a conceptually similar way. Similarly, we also established invariance across gender and across grade levels. Measurement invariance testing is, therefore, immensely helpful to increase our confidence about the robustness and validity of the S-TSRI measure. Establishing measurement invariance is an important step in the journey of validation of measures because if measurement invariance cannot be established, then a between-group difference cannot be interpreted without ambiguity as it will not be clear if this difference is due to a “true” difference on the construct of interest or to different psychometric responses to the scale items (Cheung and Rensvold, 2002).

We subsequently tested the differences between the pass and fail groups across various study variables. The pass group reported significantly higher scores on having a sense of school belonging and having a satisfactory relationship with their teachers, compared to the fail group. Those who failed reported significantly higher conflict with their teachers and significantly higher scores on aggression. Consistent with the research literature using samples from North America and Asia as would be expected, compared to students with lower academic achievement, those with higher academic achievement were more satisfied with the teacher-student relationship, had less conflict with their teachers, and more positive attitudes toward their teachers and schools (Chong et al., 2010; Sivan and Chan, 2013; Suldo et al., 2014). Interestingly, both the students from the pass and fail groups reported a similar level of instrumental help from their teachers. In this sample, from the students’ perspective, teachers did not discriminate and provided just as much instrumental help to the fail group as they did the pass group. This is a noteworthy finding that deserves some emphasis and elaboration. A positive teacher-student relationship is arguably even more vital to academically or behaviorally at-risk students. Researchers have shown that for academically at-risk samples, a positive teacher-student relationship was associated not just with students’ higher academic achievement but also students having a lower level of externalizing problems, and a stronger sense of school belonging and school engagement (Hughes and Kwok, 2007; Wu et al., 2010). Consistent with the tenets of attachment theory, for academically or behaviorally at-risk students, teachers serve as an important compensatory resource for these students.

Further evidence of the validity of the S-TSRI was established by providing evidence of satisfactory convergent and discriminant validity across the three samples: the total sample and the pass and fail groups of students. Specifically, we examined the relationships among the positive (satisfaction, instrumental help) and negative (conflict) teacher-student relationship dimensions, and outcomes, such as the students’ sense of school belonging and also their aggressive behavior in Hypotheses 3–6. The findings were all in line with previous research and in hypothesized directions with one exception for the fail group. Positive dimensions of teacher-student relationships were positively associated with each other, and inversely associated with negative dimensions (Wentzel, 2009; Cadima et al., 2015; Vervoort et al., 2015). Likewise, for outcome variables, a sense of school belonging has an inverse relationship with aggression (Cemalcilar, 2010; Duggins et al., 2016).

With respect to conflict, aligned with international research findings, conflict was found to be positively related to aggression (e.g., Sette et al., 2016) and had a weaker negative or non-significant association with a sense of school belonging (e.g., Duggins et al., 2016) for all three samples. For satisfaction and instrumental help, consistent with the literature, these were positively associated with a sense of school belonging and had weaker negative or non-significant associations with aggression for the total sample and the pass group (Suldo et al., 2014; Hagenauer et al., 2015; Sette et al., 2016). For the fail group, instrumental help was positively correlated with aggression (β = 0.15, *p* < 0.05), and this was an unusual finding. We had earlier reported similar levels of instrumental help from teachers for both the pass and fail groups of students. Despite the fail group (academically at-risk students) having higher levels of aggression and greater conflict with teachers which are consistent with international literature (Chong et al., 2010; Sivan and Chan, 2013; Suldo et al., 2014), teachers did not reduce or shy away from providing the necessary support and help to these students. The students in the fail group would very likely exhibit a different needs profile, given their greater levels of conflict with teachers and higher aggression levels. From the students’ perspective, instrumental help from teachers is likely to be assumed or expected as part of a teacher’s role, and these higher levels of aggression displayed by these academically at-risk students could be due to a variety of factors including these students having difficulties in emotional self-regulation, relationship issues at home or among peers, not all of which are necessarily teacher-related issues. It may also be possible that this correlation is directly or indirectly related to behavioral practices from other personnel besides the form teacher. Importantly, as this is a cross-sectional study, no causality is implied in this finding.

Furthermore, we performed correlation analyses between TSRI dimensions (Satisfaction, Instrumental Help, Conflict) and the English language examination scores to provide evidence for criterion validity. As would be expected, results showed that students who reported a greater satisfaction in the teacher-student relationship had higher English language examination scores in the total sample of students as well as pass and fail groups of students. Students who had more conflictual relationships with their teachers had lower examination scores for English language across all three samples. Students’ perceptions of instrumental help was unrelated to academic achievement. These findings are consistent with extant research which shows that a positive satisfactory teacher-student relationship was found to be related to students’ higher academic achievement, and a negative conflictual teacher-student relationship was related to students’ lower academic achievement (Hamre and Pianta, 2001; Wu et al., 2010).

Some limitations of the study and directions for future work would be helpful to point researchers to further work in the area of teacher-student relationships or specifically on the S-TSRI. We established measurement invariance for three dimensions in the present study: levels of academic achievement, gender, and grade level. Measurement invariance could be tested for other relevant and key dimensions not examined in the current study. For levels of academic achievement, we used students’ end-of-the-year English language examination score in the study. Future work could also use other subjects such as Mathematics. With respect to establishing convergent and discriminant validity for the current study, we only used two outcome variables, a sense of school belonging, and aggressive behavior. Additional outcome variables could be studied to provide further validity information for the S-TSRI. In the present study, only upper primary school students were included, and future studies can extend the validation work to older students. Future work can also consider examining the cross-cultural validity of the scores from the S-TSRI, as well as to report test-retest reliability for the S-TSRI. It will also be helpful for future work to examine how the quality of dyadic teacher-student relationships and its outcomes might be influenced by parents, peers, other teachers, and personnel.

In conclusion, the 14-item S-TSRI is a brief measure of the quality of teacher-student relationships viewed from the students’ perspective. Findings from this study show that the measure has robust psychometric properties, and yields scores that are reliable and valid in this large sample of primary school students from Singapore. Brief yet robust measures such as this reduce respondent fatigue and can be more efficiently administered alongside other measures in schools.

## Data Availability Statement

The datasets generated for this study will not be made publicly available. The de-identified dataset belongs to the Ministry of Education (Singapore) and data usage is guided by MOE (Singapore)’s data management policies.

## Ethics Statement

The studies involving human participants were reviewed and approved by the Institutional Review Board (IRB) of Nanyang Technological University, Singapore. Written informed consent from the participants’ legal guardian/next of kin was not required to participate in this study in accordance with the national legislation and the institutional requirements.

## Author Contributions

RA was responsible for the overall conceptualization and design of the study, and the writing of the manuscript. SO was responsible for the co-conceptualization of the study and liaising with MOE colleagues across divisions. XL was responsible for the data analysis and the writing of the manuscript. All authors contributed to the final version of the manuscript.

## Conflict of Interest

The authors declare that the research was conducted in the absence of any commercial or financial relationships that could be construed as a potential conflict of interest.
